# Cross-disciplinary intersections between public health and economics in intimate partner violence research

**DOI:** 10.1016/j.ssmph.2021.100822

**Published:** 2021-05-15

**Authors:** Meghna Ranganathan, Lori Heise, Amber Peterman, Shalini Roy, Melissa Hidrobo

**Affiliations:** a Department of Global Health and Development, Faculty of Public Health and Policy, London School of Hygiene and Tropical Medicine, Tavistock Place, WC1H 9SH, London, UK; b Department of Population, Family and Reproductive Health, Johns Hopkins Bloomberg School of Public Health and Johns Hopkins University School of Nursing, 615 N. Wolfe Street, Room E4644, 21205, Baltimore, MD, USA; c Department of Public Policy, Abernathy Hall CB #3435, University of North Carolina at Chapel Hill, Chapel Hill, NC, 27516, USA; d Poverty Health and Nutrition Division, International Food Policy Research Institute, 1201 I St NW, Washington, DC, 20005, USA

**Keywords:** Intimate partner violence, Cross-disciplinary research, Public health and economics

## Abstract

Research on intimate partner violence (IPV) has progressed in the last decade in the fields of public health and economics, with under-explored potential for cross-fertilisation. We examine the theoretical perspectives and methodological approaches that each discipline uses to conceptualise and study IPV and offer a perspective on their relative advantages. Public health takes a broad theoretical perspective anchored in the socio-ecological framework, considering multiple and synergistic drivers of IPV, while economics focuses on bargaining models which highlight individual power and factors that shape this power. These perspectives shape empirical work, with public health examining multi-faceted interventions, risk and mediating factors, while economics focuses on causal modelling of specific economic and institutional factors and economic-based interventions. The disciplines also have differing views on measurement and ethics in primary research. We argue that efforts to understand and address IPV would benefit if the two disciplines collaborated more closely and combined the best traditions of both fields.

## Highlights

• Public health and economics take different approaches to IPV research.
• Public health has a broad, multi-level theoretical lens, analysing risk pathways.
• Economics focuses on individualistic causal models and analysis of causal impacts.
• Disciplinary differences also include ethical and measurement considerations.
• Increased inter-disciplinary research will facilitate global efforts to end IPV.

## Introduction

Intimate partner violence (IPV) is a major global public health challenge with one in three women ever experiencing lifetime physical and/or sexual IPV (World Health Organization, 2021). In addition to causing physical injury and adverse health outcomes (Bacchus, Ranganathan, Watts, & Devries, 2018), IPV has been associated with adverse social and economic outcomes for women, households and communities (Heise, 2012). In the last decade, significant theoretical and empirical advances have been made (Wu, Chen, Fang, & Wan, 2020) that improve understanding of global IPV prevalence (Devries et al., 2013), underlying drivers (Yakubovich et al., 2018) and prevention (Ellsberg et al., 2015). IPV research has historically been undertaken by public health or feminist scholars, conceptualising IPV as a phenomenon driven by complex socio-ecological factors and using mixed methodologies (Heise, 1998; Jewkes, 2002). In particular, public health researchers often study complex interventions to shift individual attitudes, alongside factors at the community level (e.g., social norms condoning male authority) (Abramsky et al., 2014) or at the interpersonal level (e.g., poor communication, conflict negotiation skills; and alcohol abuse) (Dunkle, Stern, Chatterji, & Heise, 2020). However, IPV is increasingly being studied by diverse disciplines, including economics.

Economic literature on IPV emerged in the early 1990s, with an initial focus on developing and empirically testing theoretical models of the family that conceptualised how interactions between partners could lead to IPV and how economic or institutional factors affect these interactions and hence IPV (Farmer & Tiefenthaler, 1997; Tauchen, Witte, & Long, 1991). These inquiries have gradually expanded to evaluations of how diverse policies, institutions, and economic factors affect IPV, such as employment and wages (Aizer, 2010; Anderberg, Rainer, Wadsworth, & Wilson, 2016), divorce laws (Bowlus & Seitz, 2006), dowry (Bloch & Rao, 2002), or cash and asset transfers (Buller et al., 2018), with a continuing focus on the empirical estimation of quantitative relationships.

Despite the expansion of research on IPV *within* the disciplines of economics and public health, there has been little cross-fertilisation between them. A recent paper attempts to bridge the disciplinary divide between economics and epidemiology in general, by examining underlying differences in how the disciplines evaluate the impact of interventions using randomised controlled trials (RCTs) (Powell-Jackson et al., 2018). In this paper, we examine how these two disciplines conceptualise and study IPV as a social phenomenon. Our goal is to introduce practitioners and researchers to the different world views of each perspective and offer an assessment of their respective strengths and weaknesses for studying IPV. We conclude by providing recommendations for how the disciplines can be brought together to encourage interdisciplinary research that draws on the best of both traditions.

### Disciplinary differences in the theoretical approach to IPV

**Fig. 1:**
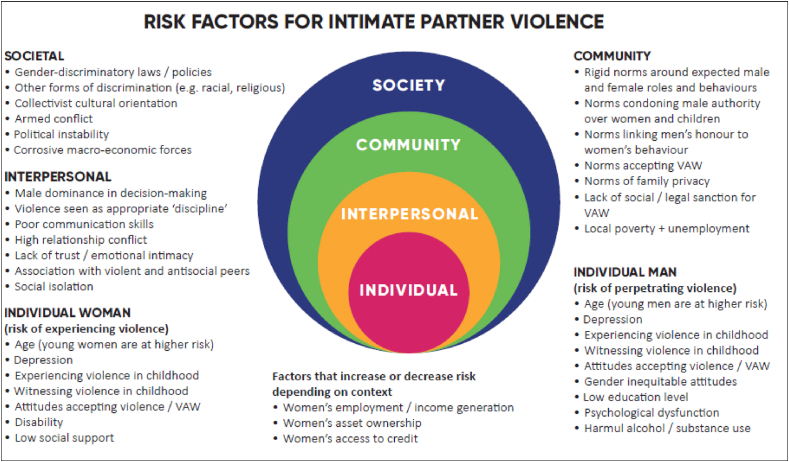
Socio-ecological framework depicting contributing factors towards IPV experienced by women.

Source: The Prevention Collaborative 2020 (The Prevention Collaborative, 2020)

Public health researchers maintain that the origins of IPV are multi-faceted and grounded in the interplay of factors operating at different levels of the “social ecology.” The discipline draws on the socio-ecological model as a heuristic tool to organize the individual, interpersonal, community and macro-level factors that combine to increase an individual's risk of perpetrating or experiencing IPV (Heise, 1998). It draws on a wide variety of biological, behavioural, psychological, and sociological theories to predict how and why different factors might contribute to the occurrence of IPV. As illustrated in Fig. 1, individuals enter relationships with their own personal genetic and epigenetic endowment, temperament, set of behaviors, developmental history and past experiences of trauma or abuse. Together, couples construct a relationship that develops its own dynamic, informed by levels of communication, conflict management, substance use, gender roles, and power differentials. The relationship and the household in which it is embedded, exists within social and material realities, including family and community norms, economic opportunities, social and legal sanctions around violence, and societal and community factors such as macro-economic forces, political stability and patriarchal ideology.

Within public health, both qualitative and quantitative research have revealed a host of factors that increase the likelihood that a particular individual in a particular setting, might perpetrate or experience IPV. Fig. 1 summarises the factors that have emerged repeatedly as linked with IPV, especially in low-resource settings. In this paradigm, no single factor or behavioural insight is necessary nor sufficient to predict IPV—although feminist researchers emphasise the central role that patriarchal beliefs, norms and social relations play in justifying and driving violence. Like heart disease, the paradigm that best explains IPV is one of “risk,” where the presence of multiple, overlapping risk factors increases the *likelihood* that IPV may occur, but it does not predetermine it. Factors also interact across levels, with some serving to potentiate or dampen the effect of others working at more proximate levels of the social ecology (Ackerson & Subramanian, 2008; Heise & Kotsadam, 2015). Public health also recognizes that clustering of IPV occurs within regions and among neighbourhoods. Understanding the source of this variation can help uncover important factors that contribute to IPV patterns.

Economists tend to conceptualise IPV within household models where individuals (men) make explicit choices about whether to perpetrate IPV within a relationship; their partners (women) make explicit choices about whether to stay in the relationship (and if they stay, how to minimize violence) (Farmer & Tiefenthaler, 1997; Tauchen et al., 1991). These choices are motivated by each individual's desire to maximize their own utility (well-being), subject to constraints. Typically for the man, IPV is linked to utility either for expressive reasons (e.g. to derive direct pleasure or to release frustration or both) (Farmer & Tiefenthaler, 1997), for instrumental reasons (e.g. to control his partner's behaviour or take control over resources) (Bloch & Rao, 2002; Eswaran & Malhotra, 2011) or for reasons of status inconsistency (e.g. to re-assert dominance when the woman's status increases relative to his own, reflecting “backlash”) (Chin, 2012). Men can also experience disutility from perpetrating IPV, due to an inherent distaste for IPV or due to an increased likelihood of the woman leaving the relationship. It is assumed that men and women derive benefits from the relationship, however, women experience disutility when IPV is perpetrated against them.

Economists' typical approach to combining men's and women's utility maximisation is through a household bargaining model based on game theory, whereby each individual's choice is the optimal response to their partner's choice. An implication is that the bargaining power of each is shaped by their “reservation utility” or “threat point” – meaning, how well off they would be if the relationship dissolved (Manser & Brown, 1980; McElroy & Horney, 1981) or if they settled on a noncooperative equilibrium (each operating independently) (Lundberg & Pollak, 1993). The threat point reflects the minimum level of utility each must achieve within the relationship to stay. Women choose to stay in the relationship if their utility within the relationship (factoring in whether the man perpetrates IPV) exceeds their utility outside the relationship; men choose to perpetrate IPV if the utility of doing so (factoring in whether the woman leaves) exceeds the utility of not doing so. A prediction is the more resources or opportunities an individual can command outside the relationship, the stronger the individual's threat point. As the threat to leave becomes more credible, women's bargaining power within the relationship increases (McElroy & Horney, 1981).

Because economic models specify how men's and women's behaviour responds to external factors, as well as to each other's behaviour, economists use models to predict how a change in a particular factor of interest, such as income controlled by women will affect IPV (Farmer & Tiefenthaler, 1997). These models are typically stylised and consider a limited number of factors and interactions between factors for tractability (McElroy, 1990). Although models have been extended over time – for example, to capture specific ways that men may use IPV to control their partner's behaviour or take control of resources (Bloch & Rao, 2002; Eswaran & Malhotra, 2011) – complex relationships between individuals and their environment often remain simplified. As a result, many economic models appear to ignore factors considered critically important to public health researchers. Empirically, these factors are either implicitly assumed to be constant or in an error term and uncorrelated with included explanatory factors.

### Differences in expertise and objectives in conducting impact evaluations

A key distinction between public health researchers and economists working on IPV is in the type of expertise they bring. Public health researchers studying IPV tend to have more prior content expertise, for instance, via working as anti-violence advocates, service providers, or prevention practitioners. Public health researchers are also more likely to work from violence-specific research centers, and as a result, have in-depth experience collecting, analysing and interpreting IPV measures alongside strong linkages with other violence stakeholders. This blending of research and practical experience is less common in the field of economics, where economists frequently pursue analytic work across a wide range of topics and settings, often on issues thought of as structural determinants of violence (e.g., poverty, education, criminal justice).

While there is significant overlap in empirical approaches, each discipline brings a unique perspective to programme evaluations. Impact evaluations in public health frequently assess the full impact of an intervention within a broader system, whereas economists tend to focus more narrowly on specific programmatic components or policy levers. Thus, public health researchers might evaluate a complex intervention that works at multiple levels to shift individual attitudes and social norms related to the acceptability of IPV, and to improve conflict management skills within couples, such as the *Indasyhikirwa* programme in Rwanda (Dunkle et al., 2020). When evaluating such interventions, public health researchers often seek to understand whether the entire package reduces IPV. Although evaluating whether multifaceted interventions collectively reduce IPV makes it difficult to isolate the specific mechanism(s) through which a programme achieves its goal, researchers seldom assume that there is a singular relationship between a behavioural mechanism and a desired outcome. Rather, the paradigm assumes any positive outcome is the product of multiple, sometimes synergistic pathways. To interrogate these pathways, researchers often collect qualitative data as another way to assess programme impacts (McLean, Heise, & Stern, 2020) and include an embedded process evaluation to track both the quality of programme implementation and unanticipated effects of the research or intervention (Stern & Nyiratunga, 2017). These elements help triangulate and interpret the quantitative findings and are especially important if the trial fails to demonstrate significant effects (or finds adverse effects).

Economists also evaluate multi-component interventions through RCTs but view their objectives somewhat differently. When possible, economists tend to prefer designs that allow estimating the marginal effect of each intervention component and quantifying the benefit of bundling components (Roy, Hidrobo, Hoddinott, & Ahmed, 2019). It is implicitly assumed that marginal benefits matter and can be weighed against marginal costs when formulating recommendations. In part, this reflects an experimental mindset that asks not only whether a programme works to affect a specific outcome, but how an existing programme can be improved (Basu, Rosenblatt, & Sep, 2020). Additionally, given the disciplinary preference for identifying structural relationships and causal drivers of behaviour, economists use RCTs to inform more narrowly construed questions of individual and household behaviour. Thus, economists may manipulate a narrow aspect of a program – for example, whether economic transfers are given as cash or in-kind – to investigate what impact this design feature has on IPV and to test hypotheses around women's control over income potentially differing by modality (Hidrobo, Peterman, & Heise, 2016). Although use of qualitative work and process evaluations to provide support for causal explanations is growing (Buller, Hidrobo, Peterman, & Heise, 2016), these approaches have traditionally been less common in economics. Finally, economists are more likely to engage in estimating the value for money of interventions, for example using cost-effectiveness or cost-benefit analysis (Ferrari et al., 2019).

### Differences in analytic approaches, data sources, and indicator construction

*Analytic approach*: In addition to using RCTs, economists use a wider range of analytic methods to establish causality in the absence of experimental variation. These quasi-experimental approaches, such as “natural experiments,” use quasi-random variation in exposure to a policy, event or intervention to establish its impact on an outcome of interest. Common methodologies include use of instrumental variables, propensity score matching, and regression discontinuity design; these approaches could be productively applied to IPV studies in public health as well. Correspondingly, economic journals prioritise studies that carefully establish causation and systematically explore all alternative explanations for a finding. This is a practice that is neither expected nor easily accommodated by public health journals, in part due to word limits. While economists are concerned with establishing causality, public health researchers are more concerned in establishing associations of multiple risk factors using specific modelling techniques for certain types of data. They also often conduct mediation analysis and use mixed-methods approaches for delineating pathways of risk.

*Data Sources*: The disciplines tend to make different assumptions regarding the relative value of self-report versus administrative data. Economists use both self-report and administrative data (police, health service, or facility data). By contrast, public health researchers rely more heavily on self-report data. Given research showing that administrative systems capture only a small subset of IPV (generally thought to be the most severe cases) (Palermo, Bleck, & Peterman, 2014), public health researchers view the bias introduced through self-reporting as less significant than the bias introduced by relying on administrative systems. Both disciplines, however, have investigated ways to maximize the validity of self-report data, with public health researchers exploring ways to maximize disclosure through question design and by enhancing privacy and safety, and economists exploring use of anonymous methods such as list randomisation (Lépine, Treibich, & D’Exelle, 2020).

*Indicators*: Differences also appear in indicator construction. In collaboration with the World Health Organisation (WHO), public health researchers have developed and validated measures for physical and sexual IPV, psychological abuse, and economic coercion (Garcia-Moreno, Jansen, Ellsberg, Heise, & Watts, 2006). These validated measures are binary indicators of different types of violence that are created from multiple behaviourally specific questions on a range of abusive acts (Ellsberg et al., 2015). Economists on the other hand are more likely to use non-standard measures. For example, some economics papers (Haushofer, Ringdal, Shapiro, & Wang, 2019) use aggregate indices for IPV using a variety of aggregation methodologies, in addition to or instead of separately analysing different types of IPV. This is in part to circumvent issues of multiple hypothesis testing, but the interpretation of aggregate indices is less clear. Economists are also more likely to conduct analysis at the intensive margin, investigating impacts on severity or frequency of IPV, instead of just binary measures of experience (Peterman, Valli, & Palermo, 2021). While public health researchers spend time at the outset validating measures of IPV via qualitative methods, economists rely more on sensitivity analysis of indicator construction and functional form at the analysis stage.

### Safety and ethical considerations

Researchers in public health have pioneered guidelines and practices to ethically collect sensitive information around IPV for safety and to minimize risk and harms. While best practice has evolved over time in response to new developments, ethical leadership remains grounded in public health (World Health Organization, 2001). Based on guidance from public health researchers, there is consensus that IPV data collection requires multiple safeguards to protect participants’ safety and wellbeing. This includes providing specialised training for interviewers, ensuring detailed informed consent and privacy during interviews, providing referral protocols, providing field staff with psychological support, and considerations around how the research should be communicated to the participant community.

Regardless of discipline, all studies on human subjects are reviewed by ethics boards nationally and internationally (as applicable). However, as many ethics review boards have minimal training in the ethics of violence, all IPV research relies at least partially on the training and expertise of researchers to self-enforce and promote ethical practices within primary research. Public health journals typically require authors to provide information about ethical components, including risks considered, any observed harms, risk to research team members, and details of the consent process. In economic journals, to date, there is no standard requirement to include such details in published papers, despite some recent initiatives calling more generally for greater transparency in ethics (Asiedu, Dean, Monica, Lambon-Quayefio, & Udry, 2021). Consequently, in practice, it is unclear whether primary research in economics adheres to similar ethical protocols as public health. The lack of reporting on ethics in economics is especially problematic as both the quality of data and the lives of women are put at risk without sufficient attention to ethical protocol. For example, evaluations comparing rates of IPV disclosure among women interviewed by enumerators with more extensive training and sensitisation, compared to those with far less, illustrate the impact of more interviewer preparation on prevalence estimates (Jansen, Watts, Ellsberg, Heise, & García-Moreno, 2004).

## Forging a cross-disciplinary path

Public health researchers and economists each bring a distinct set of strengths and methodological tools to the IPV field, and cross-fertilisation could further advance IPV prevention efforts.

Economists are increasingly conducting impact evaluations to inform IPV prevention, leveraging large-scale interventions and data collection efforts. This is a positive development for strengthening the evidence base on preventing IPV. It also presents an important opportunity for economists to join forces with public health researchers with expertise in this area, to develop collaborations that build a richer body of evidence in a number of ways. First, literature from public health demonstrates the important role of multi-level factors that are often not included in economic models of IPV. Although not every economic model needs to include all factors, it is important to consider those relevant for answering specific research questions. For example, acceptability of IPV and gender norms are typically not included in economic models of IPV, although recently economists have started to empirically examine such factors (González & Rodríguez-Planas, 2020; Tur-Prats, 2019). Their omission may limit generalisability across settings; for example, in settings where women believe they deserve to be physically punished for disobeying their husbands, interventions that increase women's bargaining power may not reduce the level of IPV they are willing to accept. Economic studies that aim to understand impact pathways for IPV might thus benefit from broadening their focus from one unified economic model to include multi-level factors that may shape impacts and mixed-method techniques that highlight the factors that are important. A broader approach would move toward including factors motivated by economic models, but also drawing upon lessons from across disciplines and methodologies (Buller et al., 2016). Building multidisciplinary teams would also improve management of the complex safety and ethical issues around collecting IPV data.

Within public health, multidisciplinary teams could also yield important benefits. For example, insights from economists could shed light on hypothesised linkages when interpreting impacts of a multi-component IPV prevention programme, such as the relationship between IPV and income controlled by the woman. Furthermore, public health research could draw from economists’ diverse approaches to establishing causality outside the context of an RCT. There may be underutilised opportunities in public health to seek out natural experiments or use quasi-experimental methods to evaluate policies or structural factors in the absence of randomisation. Within empirical analysis, methodologies to account for selection bias, as well as robustness checks for alternative explanations and analysis choices that are routine in economics, would strengthen public health approaches.

Disciplinary divides exist due to a wide range of factors, including silos in funding streams, professional networks, and expertise, as well as differences in publishing paradigms, journal rankings, and career advancement incentives. However, actions to directly address these barriers to collaboration could encourage more successful inter-disciplinary knowledge production. These may include: engaging donors that fund research on IPV to recognise the value of inter-disciplinary work, collaborating on joint funding proposals for projects and funding streams, organising workshops to encourage networking across disciplines, forming a virtual network to jointly address data quality and measurement issues, creating a shared ethical framework to underpin research, and encouraging academic programmes and journal editors in both disciplines to promote and reward mixed-methods interdisciplinary work. With these efforts, the two disciplines may jointly strengthen IPV research and also achieve larger objectives of each – for example, increasing evidence to policy translation and expanding inclusion of researchers from the global South, given distinct but complementary access to stakeholders and global networks. We thus argue that bringing the two disciplines together offers the promise of developing novel research that yields deeper insights into IPV and ultimately facilitates global efforts to end IPV.

## Funding and acknowledgements

The authors are affiliated with the Cash Transfers and Intimate Partner Violence (IPV) collaborative that is supported by funding from an anonymous donor. The funder has not been involved in the writing or submission of this research.

The authors are enormously grateful to participants from the *“Cross-disciplinary intersections between Economics and Social Epidemiology in Intimate Partner Violence research”* workshop organised by the Gender Violence and Health Centre at the London School of Hygiene and Tropical Medicine who provided ideas that led to development of this paper.

## CRediT authorship contribution statement

**Meghna Ranganathan:** Conceptualisation, Writing – original draft preparation, revisions of subsequent drafts. **Lori Heise:** Conceptualisation, Writing - substantial contribution to original draft, reviewing and editing subsequent drafts. **Amber Peterman:** Conceptualisation, Writing - substantial contribution to original draft, reviewing and editing, reviewing and editing subsequent drafts. **Shalini Roy:** Writing - substantial contribution to original draft, reviewing and editing, reviewing and editing subsequent drafts. **Melissa Hidrobo:** Conceptualisation, Writing - substantial contribution to original draft, reviewing and editing, reviewing and editing subsequent drafts. All authors approved the final draft.

## Declaration of competing interest

The authors declare no conflict of interest.
